# Producing micro-finite element models from real-time clinical CT scanners: calibration, validation and material mapping strategies

**DOI:** 10.3389/fbioe.2025.1670428

**Published:** 2025-12-17

**Authors:** Yanni Cai, Peter Zioupos, Nicholas Márquez-Grant, Basil Budair, Sarah Junaid

**Affiliations:** 1 Cranfield Forensic Institute, Cranfield University, Cranfield, United Kingdom; 2 School of Mechatronics and Biomedical Engineering, Aston University, Birmingham, United Kingdom; 3 Biomedical Engineering, School of Engineering, University of Hull, Hull, United Kingdom; 4 Royal Orthopaedic Hospital (ROH), Birmingham, United Kingdom

**Keywords:** finite element analysis, subject-specific modelling, clinical CT scanning, validation, calibration, material property mapping

## Abstract

Finite element (FE) models from living anatomical structures to produce patient-specific models offer improved diagnosis, precision pre-op planning for surgeries, and reliable biofidelic stress loading analysis. These models require the use of clinical scanners that are safe to use *in-vivo* but offer relatively lower resolution than *in-vitro* micro-CT ones. To capitalise on the clinical advantages, this route offers certain technical challenges which must be ironed out to derive a reliable validated route from scanning to *in silico* modelling. In the present study, sheep vertebrae were used to create biofidelic phantoms for scanning by using one of the latest technology high-resolution (300 micron) clinical standing scanners (HiRise, Curvebeam). Geometric information was used to produce FEA models (Abaqus/CAE), which were then validated under compression loading in the lab. The main challenges had to do first with reading and converting the scan data from voxels to material property assignment for each FE element, which was performed by using a number of different conversion equations from the literature, and second, to a lesser degree, with the minor challenges of seeking convergence and refining the boundary conditions. The fit between the model and the experimental results was best for two equations from the literature, while others were less reliable. The selection of the most suitable and universally applicable material conversion equation is significant because it can streamline the route to produce scanner to computer patient-specific models, and make these widely available and ultimately more easily immediately obtainable post-scans. Some known clinical examples highlight the potential use of this methodology for situations where loading and unloading configurations are equally challenging for modelling (i.e., standing CT scans of feet), and this paper discusses the importance of the approach for such examples. Unlike previous studies using micro-CT or non-clinical setups, this work validates a real-time, weight-bearing CT-based workflow for biomechanically consistent finite element modelling.

## 1 Introduction

Finite Element Analysis (FEA) models derived from CT scans offer high geometric fidelity, good anatomical accuracy because they capture specific features, and realistic representation because they do not use idealised or averaged anatomy. When the scans are from *in-vitro* lab scanners the resolution can be indeed very fine at the micron level scale and these give rise to micro-finite element models which offer unprecedented structural fidelity and analysis at the micromechanical level. However, the high resolution requires X-ray energy input which exceeds current safety limits and therefore these micro-CT scanners are not customary used in clinical practice but occasionally in animal or experimental *in-vitro* research. Clinical scanners can be used safely in the clinical setting, but the trade-off is the limited lower resolution that they offer. Nevertheless, they have a wide range of advantages, such as: (i) they can be used in conjunction with pre-op planning and setting treatment strategies to suit specific patients; (ii) offer ease of access and for routine inspection in hospitals; (iii) are non-invasive; (iv) they can produce and store 3D data scores which can be repurposed for other analysis and used in automated or semi-automated workflows to further the benefits to treatment for specific patients (Guha et al., 2022; Lei et al., 2020; Koitka et al., 2021; Zhu et al., 2023; Watanabe et al., 2023; Caprara et al., 2021).

With continuous advances in scanning technology, the resolution of modern clinical scanners is improving to the point that it is now feasible to expect that FEA models with clinical scanner data will eventually reach the high level of geometric refinement and accuracy offered by micro-finite element models (albeit at the expense of high computational cost) (Jones and Wilcox, 2007; Watanabe et al., 2023). Finite element models of living anatomical structures are therefore valuable patient-specific tools for improved diagnosis, precision pre-op planning for surgeries, and reliable biofidelic stress loading analysis (Zhu et al., 2023). In cases where reconstructive surgery changes the geometry of the anatomical feature, the FEA model tracks changes in stress/strain distribution due to surgical intervention (Lei et al., 2020). Anticipating the after-effects of the surgery allows a more targeted, effective, and efficient design and execution of the surgical procedure itself.

Unlike previous studies using micro-CT or non-clinical setups, this work validates a real-time, weight-bearing CT-based workflow for biomechanically consistent finite element modelling. However, real-time *in-vivo* clinical CT scanners are still a relatively new technology. To date, no published data have validated their use in conjunction with FEA models and reconstructive surgery planning (Koitka et al., 2021; Zhu et al., 2023; Watanabe et al., 2023), or have validated the use of clinical, standing CT-based finite element models against experimental data, and material conversion equations have not been compared in such a context. Moreover, to capitalise on the clinical advantages, this route offers certain technical challenges (Jones and Wilcox, 2007), which must be resolved to derive a validated and reliable workflow from scanning to *in silico* modelling. These challenges include the material properties conversion and assignments from the scan voxels to the FEA model elements and the usual issues of convergence (optimum element size) and boundary conditions. In the present study, sheep vertebrae were used as good examples of chunky cancellous bone samples that offer a wide range of densities (and consequently greyscale scan maps) and which can be easily shaped into specimens for material testing *in-vitro*. The vertebrae were scanned with a latest technology high-resolution (300 micron) clinical standing scanner (HiRise, Curvebeam), FEA models were produced, and mechanical test results were used to validate the model. The research hypothesis was that based on the data in the current literature and other supporting software a valuable streamlined process may be found to derive patient-specific clinical models from scanner to computer. In the future such models are evidently effective tools to plan, reconstruct, simulate, and carry out bespoke surgery, which is patient specific and hence achieve better outcomes. Moreover, and in the future, this methodology will potentially also be integrated with robotic surgery.

## 2 Materials and methods

### 2.1 Samples

**FIGURE 1:**
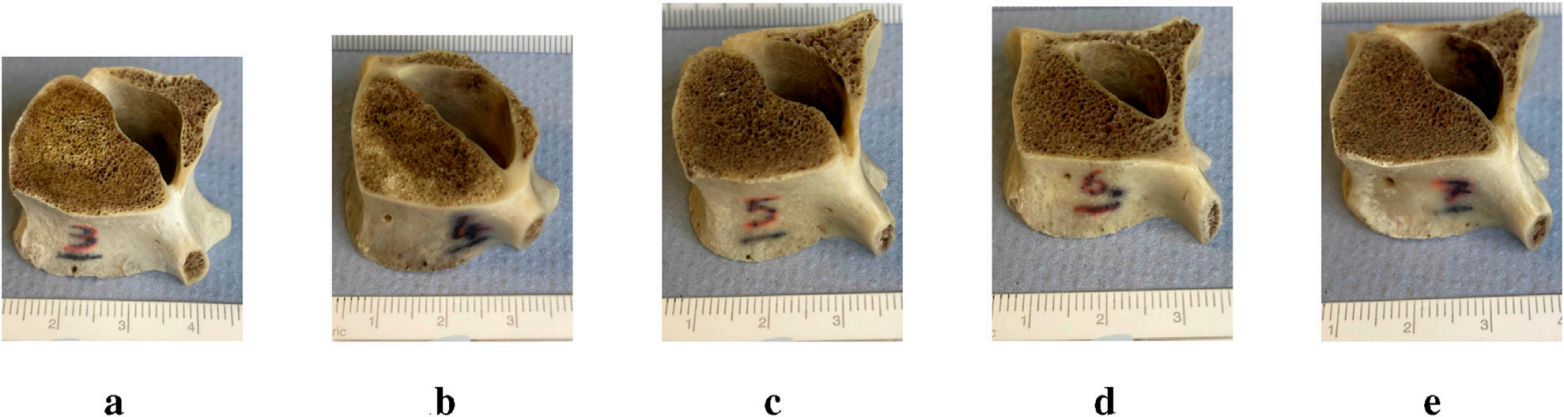
Vertebral samples with scale: After preparation, the sample heights were **(a)** #3 = 20 mm, **(b)** #4 = 17.5 mm, **(c)** #5 = 15.2 mm, **(d)** #6 = 20.6 mm, **(e)** #7 = 19.5 mm.

Seven sheep vertebrae (*Ovies aries*) originating from the lumbar region of the same mature non-pathological animal were obtained from the local abattoir and dried, cleaned and polished to have two parallel flat surfaces for compression tests. In this study, ovine vertebrae were used as an analogue of human bone (Banstola and Reynolds, 2022; Wilke et al., 1997). Ovine and human bones have been shown to have no statistical differences in the compression modulus of cancellous bone. Moreover, human and ovine bones have similar cortical and cancellous structures at the microscopic level and similar shape and size similarities, when it matters at the whole bone level. The more compelling evidence that advocates their use instead of human bone is the extensive use of sheep as an animal model for full-size implant design experimentation and accreditation, which is nowadays widespread (Datta et al., 2006; Mageed et al., 2013). Samples from the lumbar region (n = 7) were used, the first two samples were used for the set up and preliminary trials and samples n = 3–7 for testing and modelling (Figure 1).

### 2.2 Non-destructive mechanical testing

Five of the sheep vertebrae were non-destructively tested using a servohydraulic materials testing machine (Dartec series HC 25) in single-axis compression loading. Mechanical testing was performed in the biomechanics lab at the Shrivenham campus of Cranfield University, UK. Before compression testing, ultrasound gel was applied to the contact surfaces to minimise any friction in the platens. The force/displacement response of the samples was measured and recorded. The compression tests were carried out in displacement control mode, with cyclic loading of 2 Hz up to 0.8% of the total height of each sample tested (Figure 1). This ensured a strain limit (macroscopic for the whole sample) which was less than the bone yield strain, which has been reported to be ∼ 1% of the total length of a sample (Morgan et al., 2019).

### 2.2.1 Machine compliance

Machine compliance was estimated before testing the actual samples to account for the displacement that occurs in the grips, the frame, and the load cell of the testing rig. Machine compliance needs to be taken into account and the displacements it introduces need to be subtracted from the total recorded displacement so that the true displacement experienced by the sample is derived. The machine compliance was estimated to be 0.0225 mm/kN (Supplementary Appendix A).

### 2.3 FEA simulation

Finite element analysis (FEA) was performed using Abaqus/CAE (v.2019, Simulia, Providence, Rhode Island, United States).

#### 2.3.1 CT scans

**FIGURE 2:**
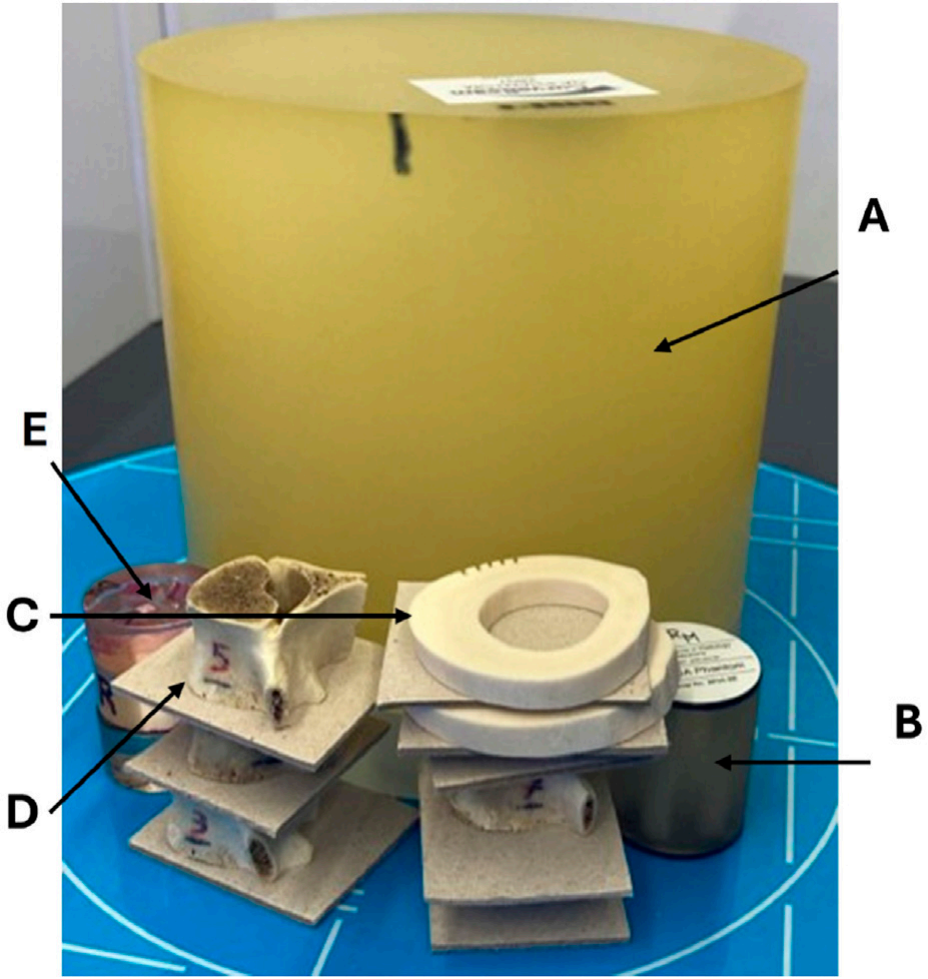
Real-time clinical scanning in the HiRise scanner in the presence of the two industrially supplied calibration standards: one for the HiRise scanner **(A)**; and the QRM HA (QRM, Lörracher Strasse 7, 79115 Freiburg, Germany) for the micro-CT **(B)**. Details for the two commercially supplied calibration phantoms are in Supplementary Appendix B. A range of bone samples were also on the same scan: cortical bone rings from mid-diaphysis of a cow femur **(C)**; the five sheep vertebrae **(D)**; and a laboratory made standard made of a variety of mammalian bone tissues for a range of mineral contents from 95% to 40% weight/weight (from Tympanic bulla to Antler bone) **(E)**. The range of all these samples was used in the same scan, so the density values and other scanning characteristics would be truly comparable under the same scanning parameters.

The 3D models used to simulate the compression test were created by segmenting the cone beam computed tomography (CBCT) scans. The five sheep vertebrae were scanned using a HiRise scanner (CurveBeam, 0.3 slice thickness, 0.3 voxel size, voltage 100 kVp and exposure 8.7 s), as seen in Figure 2, which is a modern high resolution *in-vivo* clinical scanner that is used at the Royal Orthopaedic Hospital Birmingham (UK) in collaborative projects with our lab to scan for pathological conditions such as flatfoot and planning reconstructive surgeries. The images were in Digital Imaging and Communications in Medicine (DICOM) format.

#### 2.3.2 Mesh convergence study

Before starting the FE analysis of the 3D models of the five bone samples, a mesh convergence study was carried out to refine the mesh size required to produce an output with sufficient precision without taking excessive computational time. The vertebrae had approximately the same measurements and were loaded in the same way; it was decided to apply the resulting mesh size from the mesh convergence study carried out on one sample (vertebra #3) to the remaining samples (vertebrae #4, #5, #6 and #7).

Vertebra CT scan #3 was segmented using the free software 3D Slicer (v.5.0.3), thresholding the mask between 100 HU and the maximum value, applying a wrap solidify tool to create a single solid segment from the outer surface, exporting it as a model and finally saving the model as a Wavefront Obj (.obj) file. The surface mesh was then improved through the free software MeshLab (v.2021.10) by simplifying, reconstructing, and remeshing to remove duplicate faces and vertices, t-vertices, unreferenced vertices, and repairing non-manifold edges. The surface mesh was saved again as a Wavefront Obj (.obj) file that can be opened by Solidworks (Student Edition, v.2022-2023, Dassault Systèmes, Vélizy-Villacoublay, France) where the thicken tool was used to create a solid model and saved as a STEP file (.step). The STEP file was imported into Abaqus for 3D meshing and FE analysis.

**FIGURE 3:**
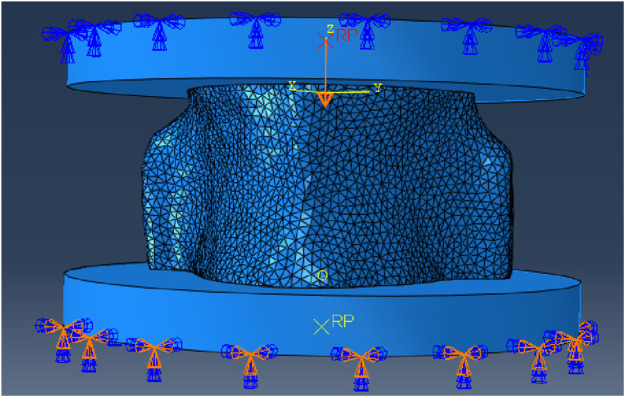
Simulation setting for the mesh convergence study showing boundary conditions to simulate the experimental mechanical test.

Boundary conditions were applied to the 3D vertebrae models to simulate the experimental mechanical test (Figure 3). The bottom pad was fixed and the top pad was loaded with a higher loading force of 2500 N (Dreischarf et al., 2016; Schafer et al., 2023). This higher loading force was chosen to go beyond the testing load used in the experimental tests and was only used for the mesh convergence step and with restricted rotation. To minimise interface problems, the upper and lower sides of the vertebrae were artificially smoothed so that the vertebrae and loading pads were fully in contact with each other. The contacting surfaces were not glued together because without a compressive force applied to one pad while the opposite pad remained fixed, the three parts would separate. A Young’s modulus of 15,000 MPa and a Poisson’s ratio of 0.3 were also applied to the vertebra model, with the loading top and bottom pads being discrete rigid bodies. This material properties were chosen because bone’s Young’s modulus has been found to be between 15,000 and 20,000 MPa with 0.3 as Poisson’s ratio (Morgan et al., 2019). A friction of 0.1 was applied to the contact between the vertebra sample and the pads and the contact between the sample and the pads was defined as “hard” which means that the surfaces in contact were not going to penetrate each other.

The two identical loading pads (50 mm in diameter and 5 mm in depth) had 133 nodes, with 130 linear quadrilateral elements (R3D4) and 2 linear triangular elements (R3D3). The sample model, instead, was meshed with linear tetrahedral elements (C3D4). The choice of using linear tetrahedral elements rather than quadratic tetrahedral elements (C3D10) was made because this simulation is a simple compression test with negligible tensile stress and bending, and therefore choosing quadratic elements would only make the analysis much more complex and time consuming, with negligible improvements in accuracy. Other types of mesh, such as hexahedral meshes were not considered because, although it seems a reasonable choice in this case, it would lower considerably the resolution of the analysed object, since it is more difficult to create complex geometries with hexahedral meshes.

**FIGURE 4:**
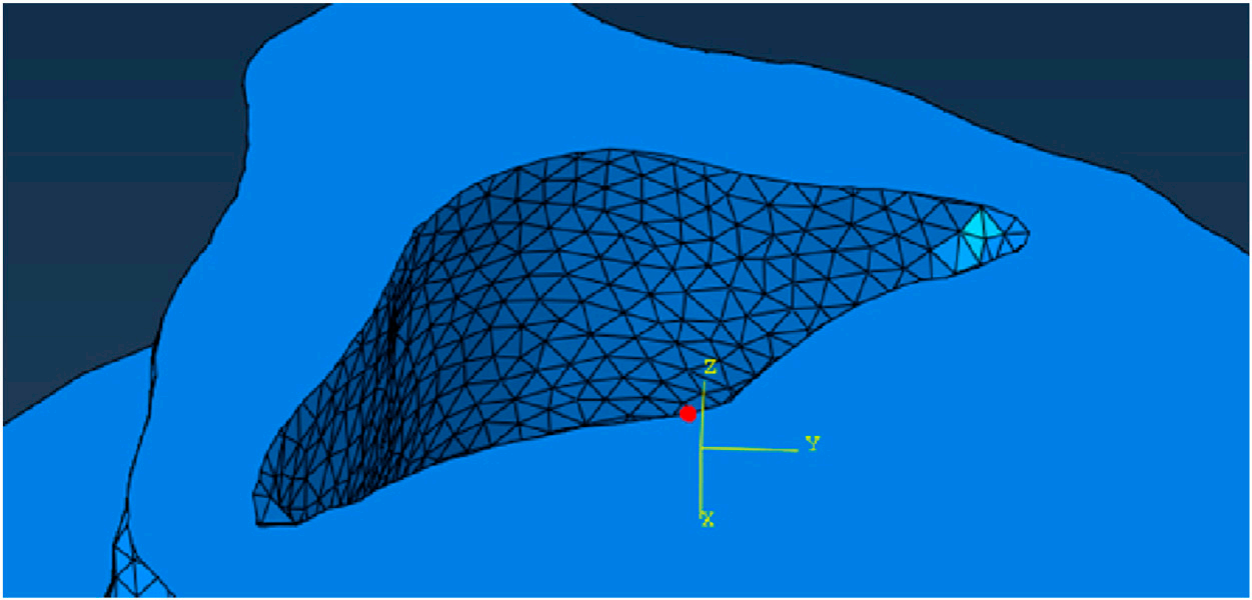
Node where the parameters were read.

Table 1 shows the results of the analyses on a node close to the centre of mass of the model (Figure 4) with different mesh sizes in percentage difference compared to the mesh with the smallest size considered (0.3 mm), which is also the voxel size of the CT scans. The parameters compared were the minimum principal strain, the displacement in the z direction, and the von Mises stress. The maximum principal strain was not considered because the model had negligible resultant tensile strains as the sample was being compressed. The mesh size that took an acceptable amount of time to analyse and had the least percentage difference from the results of the model with 0.3 mm mesh size was chosen, which was the model with 0.7 mm mesh size.

**TABLE 1:** Mesh sizes, percentage difference compared to the finest mesh of minimum principal strength, displacement in the z direction and the von Mises stress and time taken. Chosen size highlighted in bold (0.7 mm).

Size (mm)	N° nodes	N° elements	$\epsilon_{\min}$ (%)	$\delta_{z}$ (%)	$\sigma_{\mathrm{vonMises}}$ (%)	Time (s)
4	12293	58165	1.25	3.71	0.26	66
3	12817	61228	31.22	27.65	0.14	75
2	14181	69126	2.93	4.33	0.13	72
1	19909	100784	5.49	6.41	0.24	121
0.9	21136	107202	15.44	16.13	0.20	133
0.8	23378	118791	12.25	9.60	0.13	148
**0.7**	**33185**	**171246**	**2.95**	**4.37**	**0.18**	**202**
0.6	61396	324605	21.24	17.86	0.14	391
0.5	105892	571979	9.84	42.36	0.30	914
0.4	156775	855528	8.98	6.54	0.19	1590
0.3	287600	1586289	0.00	0.00	0.00	4213

Chosen mesh size is highlighted in bold (0.7 mm).

#### 2.3.3 Model reconstruction using Medtool®

3D vertebral models were created with Medtool® (v.4.6, Dr. Pahr Ingenieurs e.U., Austria), a software that allows the creation of FE models from CT scans with material properties (Young’s modulus E and Poisson’s ratio ν ) applied to each element of the model, derived from the greyscale value of the CT scans. Carter and Hayes (1977), as early as 1977 published a relationship between CT greyscale values, density, and Young’s modulus.

**FIGURE 5:**
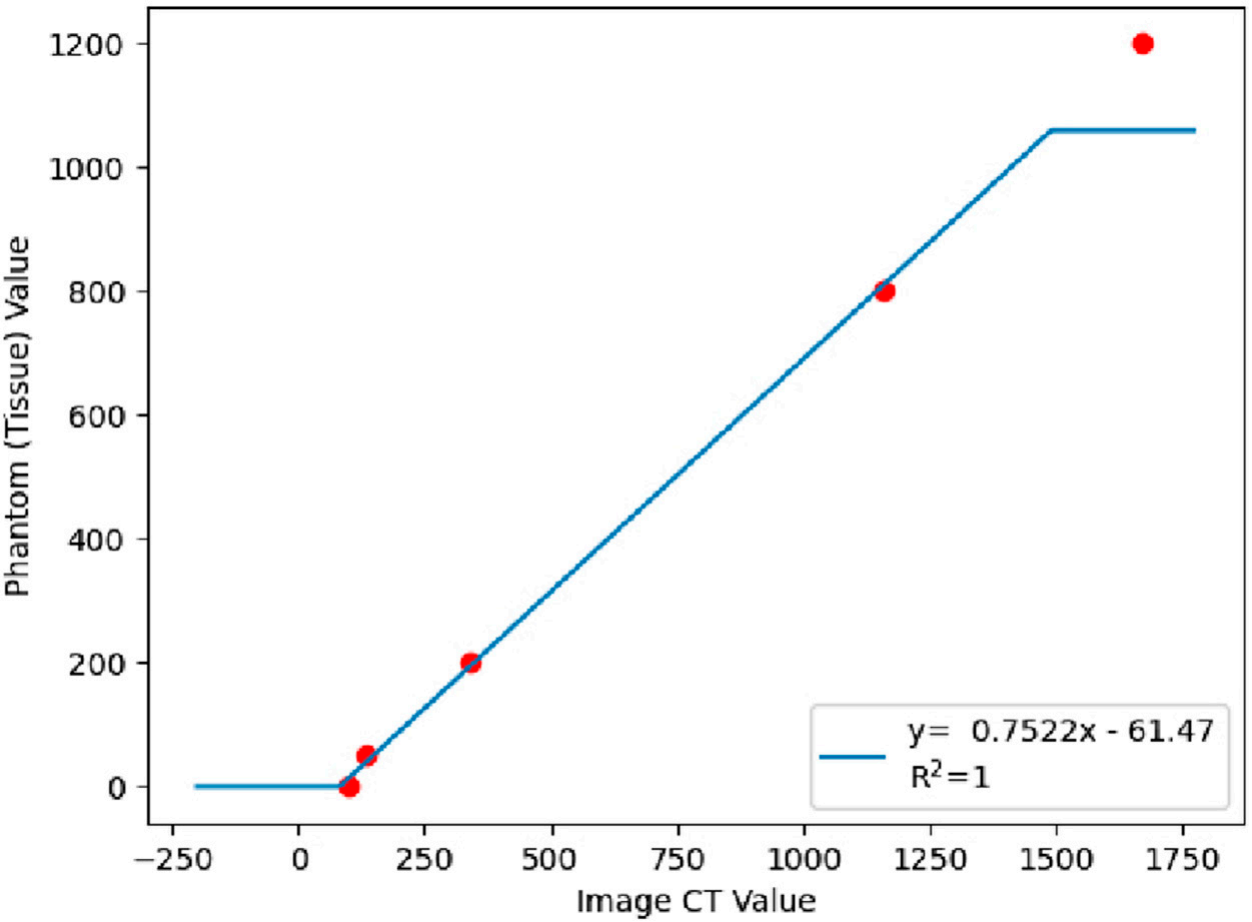
Calibration curve that shows the delimitation of the tissue between 0 and 1060 mg HA/cm^3^ to ensure that only the properties of bone material have been included in the FE model.

Medtool® is a script-based software with very little user interface. It works mainly with MetaImage MetaHeader files (.mhd); hence the CT images were first converted from DICOM to mhd format. After cropping the images to reduce their size, the CT scans were calibrated to apply the greyscale-density-Young’s modulus conversion, using the QRM HA phantom and delimiting the tissue densities between 0 and 1060 mg HA/cm^3^ (Figure 5) to ensure only the properties of bone material have been included in the FE model.

**FIGURE 6:**
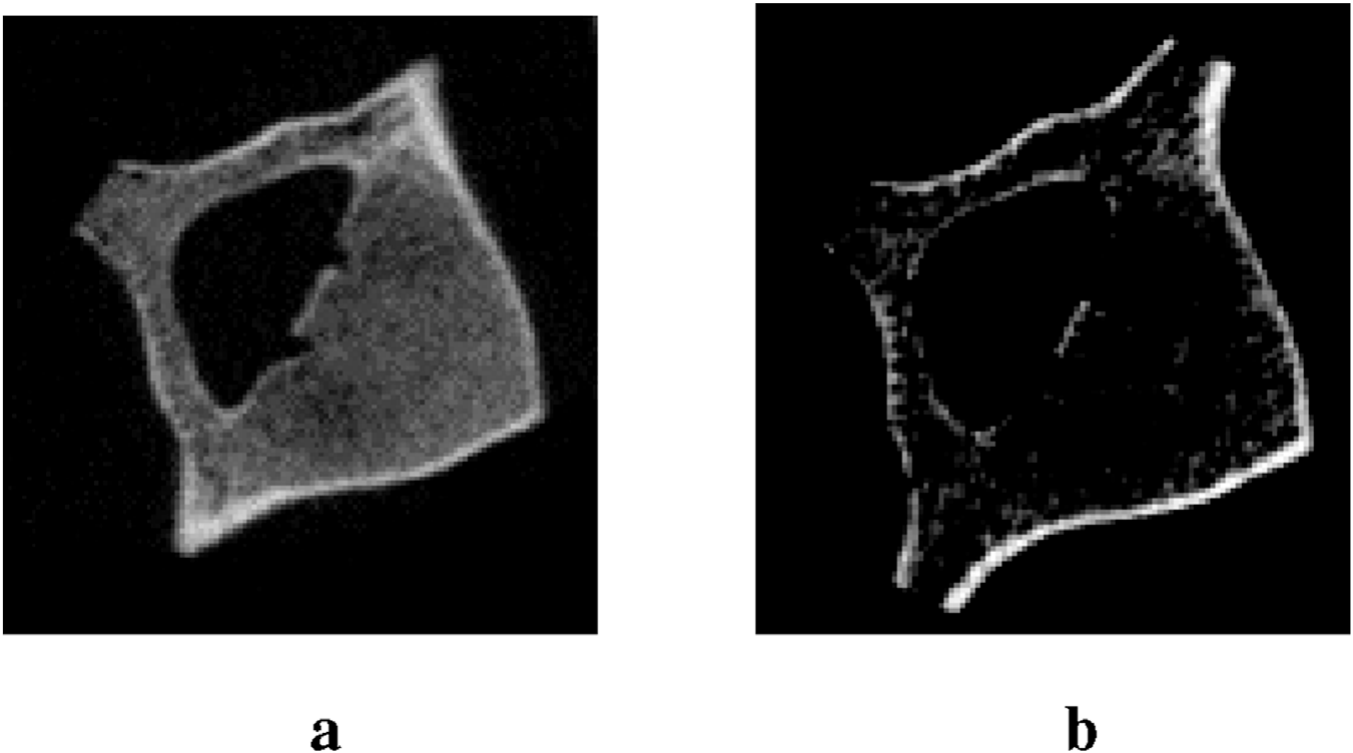
CT scans of vertebra #3 showing **(a)** scans before calibration and **(b)** scans after calibration.

The calibration using the QRM HA phantom drastically changed the appearance of CT scans (Figure 6) because the resolution of the CT scanner probably did not allow the capture of the trabecular bone with more definition, which lowered the greyscale values of the vertebral trabecular bone. If the resolution of the CT scanner was not high enough to capture the trabeculae alone, each voxel of the scan represents an averaged greyscale value of both bone and residual fat and marrow around the bone trabeculae, both of which possess considerably lower greyscale values than bone. This means that these values were not considered bone anymore; hence, they were cut off when CT scans were delimited to include only bone material.

CT scans were segmented using a MedTool® bridge with free 3D Slicer software. A threshold of 100 HU was applied to the maximum value and a wrap solidifying tool was used to make a solid image from the outer surface. Then, in Medtool®, a script was used to create a solid mesh and a mesh size of 0.7 mm was applied.

The bone volume fraction (BVTV) of the model was calculated by defining a background grid (with a distance of 2.5 mm) of cubic hexahedral elements on the CT image (Pahr and Zysset, 2009) and computing the BVTV by dividing the average greyscale value at each spherical volume (diameter of 5 mm) centred at each intersection of the background grid by 1060, so that all densities were always between 0 and 1. The isotropic material cards were defined by a Poisson’s ratio (ν) of 0.3 and the power law determined by Equation 1 was applied, with default parameters from Medtool® (Pahr, 2024). The output was an Abaqus input (.inp) file.

$$E=10,000\cdot \rho^{2}$$ (1)

#### 2.3.4 Model reconstruction using Materialise

Materialise Mimics (v.26, Materialise, Leuven, Belgium) and Materialise 3-matic (v.18, Materialise, Leuven, Belgium) are two software packages that are part of the Materialise suite and were also used to create other 3D models of the five vertebrae samples with different material cards applied to them, to examine which simulation results were the most similar to the experimental results.

After loading the CT images on Mimics, a threshold of −400 HU and the maximum value were applied to the images and a smart fill tool was used to fill any holes inside the vertebra model. If needed, a brush was also used to close any remaining gaps. A wrap and a smooth tool were also used before exporting it in 3-matic, which is specialised in creating and editing 3D models with volumetric meshes. Hence, a uniform volumetric 0.7 mm mesh was applied to the model before returning to Mimics to assign materials to the elements according to the greyscale of the CT scans.

The equations used were defined by Rho et al. (1995), who defined the material conversion equations for cancellous bone of the lumbar spine of human subjects, by developing regression equations, correlating its Houndsfield values when scanned in water with its wet weight and its elastic properties estimated using transmission ultrasonic technique. Firstly, the equation to convert the greyscale values to density (Equation 2) was used with Poisson’s ratio (ν) as 0.3.

ρ=47+1.122⋅HU (2)

Three different equations from Rho et al. (1995) were used to define E in the superior-inferior direction to produce three models for each sample for comparison (Equations 3–5).

E=−349+5.82⋅ρ (3)

$$E=0.63\cdot \rho^{1.35}$$ (4)

E=−373.822+5.954⋅ρ (5)

The models were saved as Abaqus input files (.inp) so they could be read in the FEA solver.

#### 2.3.5 FE boundary conditions

The Abaqus input files (.inp) of the models were directly imported into Abaqus, already meshed and with material properties applied. A problem surfaced when the two rigid pads were added to the simulation, and it was noticed that the upper and lower surfaces of the vertebrae models did not adhere perfectly to the surfaces of the top and bottom pads, which created problems while solving the simulation, and they also could not be made artificially flat, like in the mesh convergence study model. To avoid the interface problem altogether, similar to creating a discrete rigid part, a rigid body constraint was created in the interaction module along the upper and lower surfaces of each vertebra, which allowed one to constrain the motion of that part of the model to the motion of a reference point (ABAQUS Inc., 2009). Hence, a reference point was defined on the upper surface of the vertebra and along the z -axis of the volumetric centroid, and a rigid body constraint was made to apply the displacement, and a reference point was defined on the lower surface of the vertebra and along the z -axis of the volumetric centroid, which was also a rigid body constraint to apply the fixed boundary condition. This eliminated the need for contacting bodies.

## 3 Results

### 3.1 Main results

**FIGURE 7:**
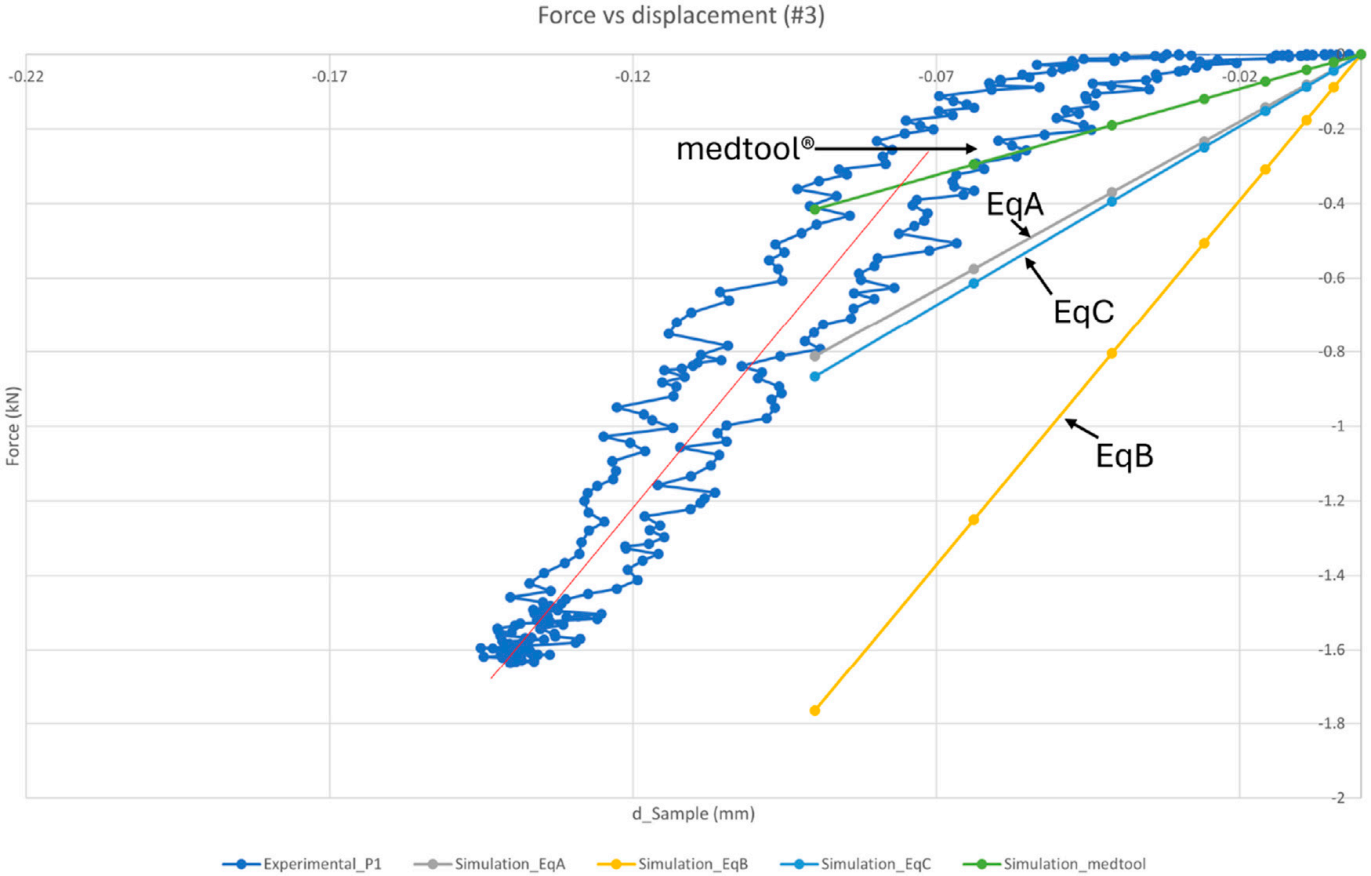
Force-displacement graph of sample #3, shown as a typical result of the actual load/displacement data (blue), together with the simulation results of Equation 3 (A-grey), Equation 4 (B-yellow), Equation 5 (C-light blue) and using Medtool® (green). The red line indicates a least squares regression of the stiffness of the experimental data in the linear elastic region. The experimental loading response was viscoelastic (showing a loop) and with an initial non-linear ‘toe’ region (for loads below 200 N) where the response became progressively stiffer before stabilising into a linear dynamic response for loads between 300 and 1600 N (for this sample). The non-linear region is due to the asperities and contact effects between the platens and the flat faces of the sample, the computer simulations in contrast account for these effects and model the idealistic case where the *in silico* model structure is loaded from zero load-zero displacement. The validation, therefore, is for comparison of the computer simulated stiffness values -vs- the reproducible linear elastic behaviour beyond the contact effects region. In this simulation, the experimental stiffness (red) was 19.6 kN/mm and the simulation stiffness (B-Equation 5) was 19 kN/mm within 3% of the experiment value.

The experimental non-destructive displacement-led compression test was carried out at 2 Hz cyclic non-destructive loading and three cycles were recorded to derive the dynamic stiffness of each of the five samples in the linear region. Each load/displacement curve showed an initial shoulder (non-linear increase in the load), which was due to the contact effects with the platens and asperities between these and the sample. Once the linear region was reached, the stiffness of the sample was measured. Different maximum displacements were used for each sample due to their different heights and different maximum loads were reached, as shown in Table 2. The force-displacement graph of sample #3 (Figure 7) and graphs of samples #4, #5, #6, and #7 show a typical cycle from the experimental test with a red line of best fit as a comparison to the FE simulations. Of the three experimental cycles, only the first complete cycle was shown to avoid graph cluttering. This decision was based on the similarity across all cycles, where the inclusion of the second and third cycles would not contribute any further information.

**TABLE 2:** Summary of samples’ displacement and related reaction forces.

Sample #	Max. Displacement (mm)	Reaction load (kN)	Act. Displacement (mm)
#3	0.14	1.6	0.09
#4	0.18	0.6	0.13
#5	0.22	1.5	0.13
#6	0.16	1.7	0.12
#7	0.16	1.8	0.11

The simulations were displacement-led and the actual displacements were applied to the vertebra models at a reference point defined along the z -axis of the volumetric centroid of the model on the top surface of the vertebra. Figure 7 shows the results of the simulations of the model with the material properties derived using Equations 3–5 (Rho et al., 1995) are depicted in grey, yellow, and light blue, respectively. The results of the simulation of the model using the Medtool® equation (Equation 1) are shown in green. The simulation models give comparable results to the compression test and also behave similarly between them across the five samples, with the Medtool® model having the softest material properties, then the models created using Equations 3, 5 being slightly stiffer, and finally the model created using Equation 4 having the stiffest material properties among all models.

Looking at the experimental datapoints, it can be seen that the five neighbouring vertebrae behave similarly among them (Table 2), having approximately a maximum displacement between 0.15 and 0.20 mm, which requires a reaction load between 1.5 and 2 kN to reach these displacements. The only vertebra that behaved differently is sample #4 that, in order to reach a displacement of 0.18 mm, needed a reaction load of “only” 0.6 kN, which is less than half of the reaction force needed for the other vertebrae to reach a similar displacement, making this bone material the softest among all other samples.

**FIGURE 8:**
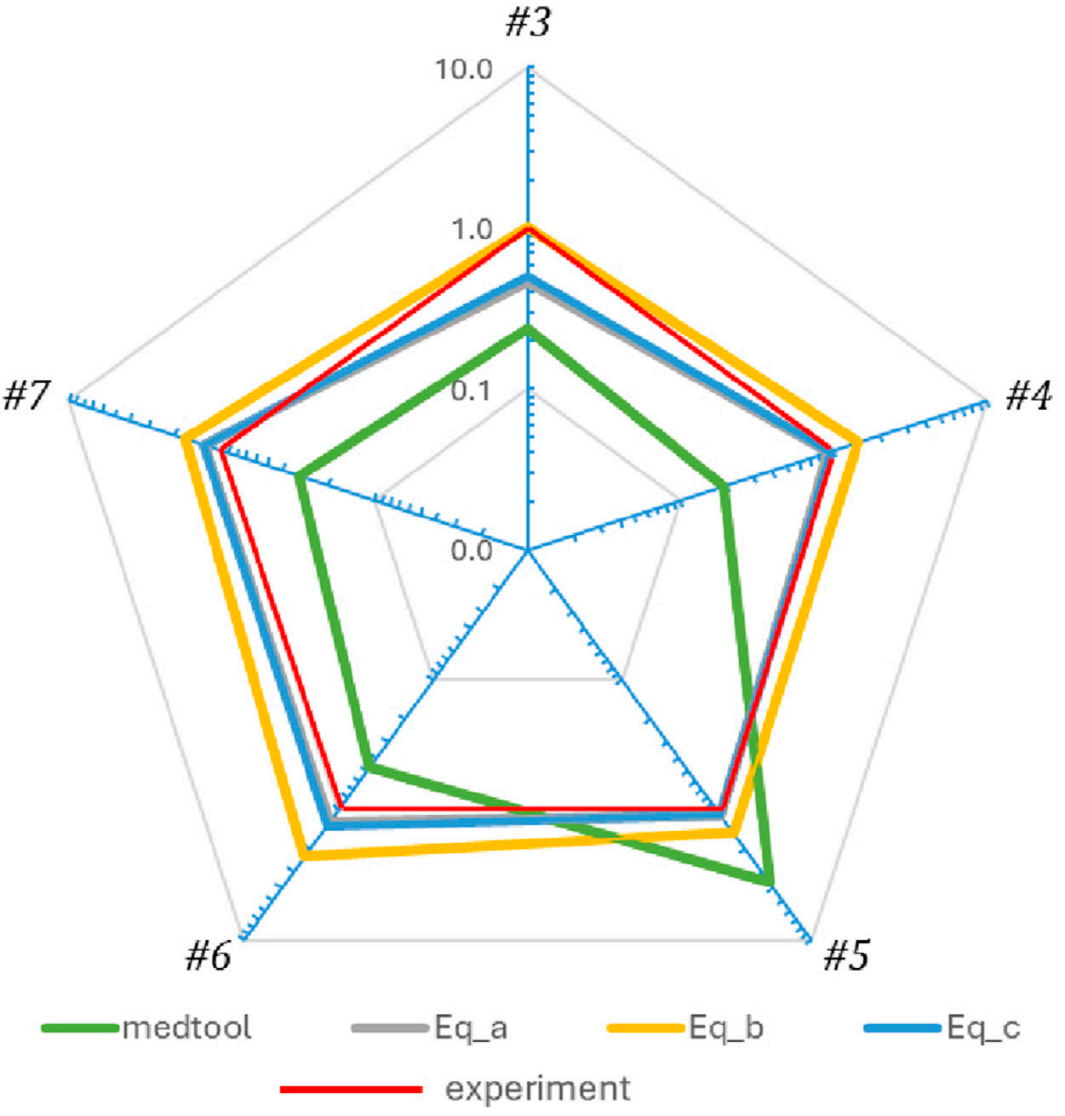
Radar graph comparing the results of the simulation model with the experimental results (red) as a ratio of experimental stiffness over simulated model’s stiffness. Lines in the same colour key code as in Figure 7. In summary, (i) Equation 3 (grey) and 5 (blue) estimated accurately the stiffness of sample #4 and overall quite well the stiffness of four out of five vertebral samples; (ii) Equation 4 overestimated the stiffness of four out of five vertebrae by as much as max 30% and accurately estimated the stiffness of just one sample #3 (example shown in Figure 7); (iii) Medtool® underestimated the stiffness of four out of five vertebrae and over-estimated the fifth by more than 50% (the absolute error was between 50% (least) and 270% (max) in all cases).

Figure 8 shows a comparison of the stiffnesses of the four simulation models for the five samples according to their similarity to the stiffness of the experimental model. If the line corresponding to a particular simulation model is at or near 1 (red line), it indicates that the ratio between the stiffness from that simulation model and the stiffness of the experimental model is 1, thus the two stiffnesses are similar. If the line is closer to the centre than 1, it means that the simulation model is less stiff than the experimental one. Instead, if the line is further away from 1, it means that the simulation model is stiffer than the experimental model. For example, in the case of model sample #4, the grey and light blue lines, which represent the simulation models created using Equations 3, 5, are at 1, showing that the model accurately predicted the experimental stiffness for this sample. Instead, the yellow line (model creation process that uses Equation 4) indicates a stiffer model, while the green line (which represents Medtool®) indicates a softer one.

Simulation models of samples #6 and #7 have a very similar order of predicted stiffness values, with Equation 4 being the stiffest and the Medtool® model the softest. For samples #6 and #7 the order of predicted stiffness values from the 4 different model process routes was the same (yellow-Equation 4 >grey-Equation 3 >blue-Equation 5 >red-experiment >green-Medtool®) For samples #3 and #4 the order of predicted stiffness values from the 4 different model process routes was also the same but different (yellow-Equation 4 >red-experiment >grey-Equation 3 >blue-Equation 5 >green-Medtool®) with Medtool® being again softest. And sample #5 is the one whose stiffness was underestimated by all 4 model processes with Medtool® overestimating the stiffness value by up to 2.73 times. The fact that adjacent samples on the spine behave similarly (#3 is similar to #4, then an inversion of behaviour at #5 and then again #6 behaves similarly to #7) and that the trend changes gradually as we move from L3 to L7 vertebrae may be a strong indication that the underlying geometry and microarchitecture also affects and interplays with the modelling process in a way that is sample-specific.

### 3.2 Boundary condition effects in simulation values

**FIGURE 9:**
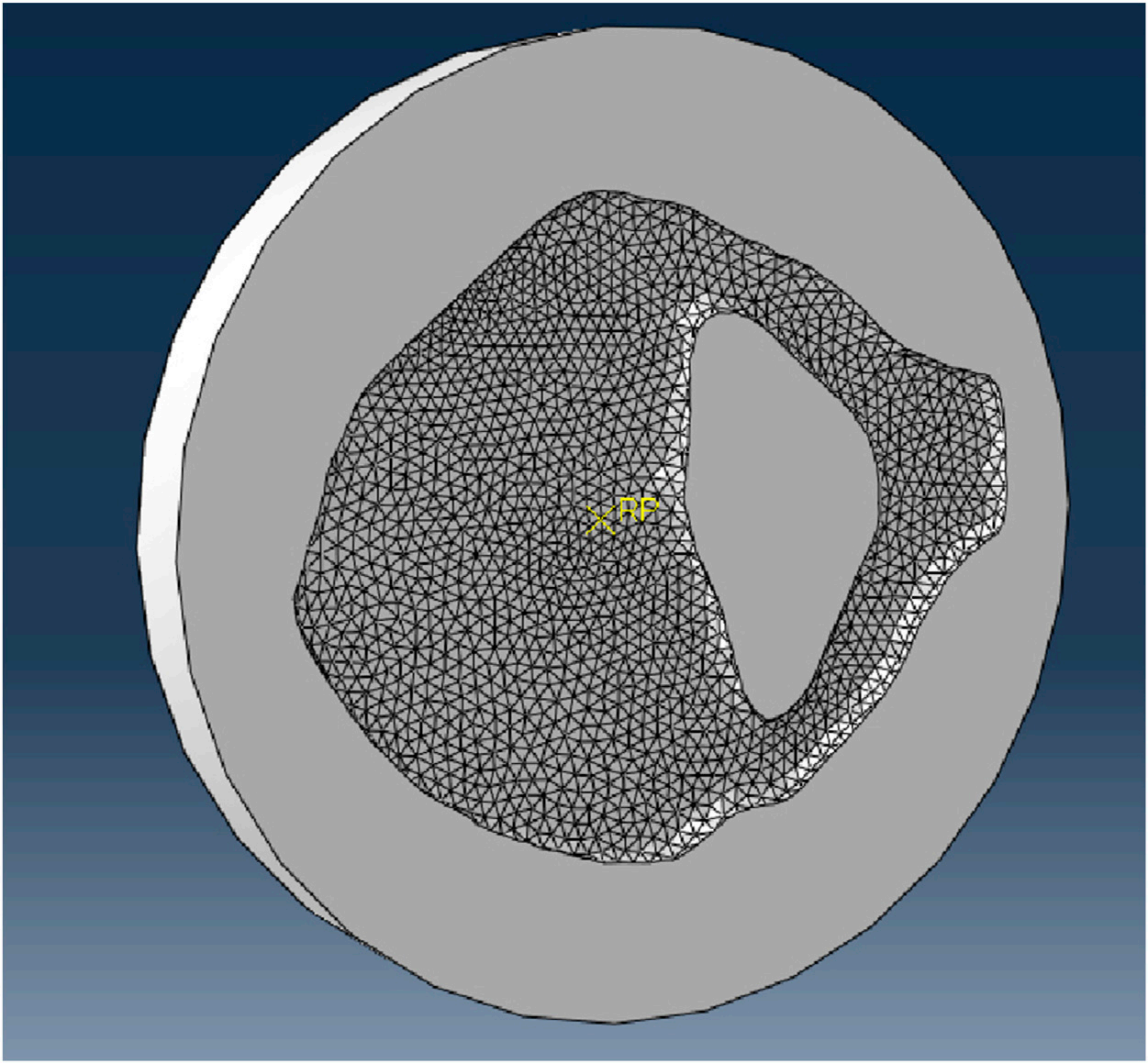
Example of a top pad in which the profile of the upper part of the vertebra has been cut.

The choice of how boundary conditions were applied was examined in more detail, and different applications of boundary conditions were identified. Instead of artificially smoothing the upper and lower surfaces of the vertebra sample was discovered that the shapes of the upper and lower surfaces of the vertebra could be cut out of the upper and lower pads (like in Figure 9). Consequently, a more detailed analysis of the implications of this modification was conducted.

**FIGURE 10:**
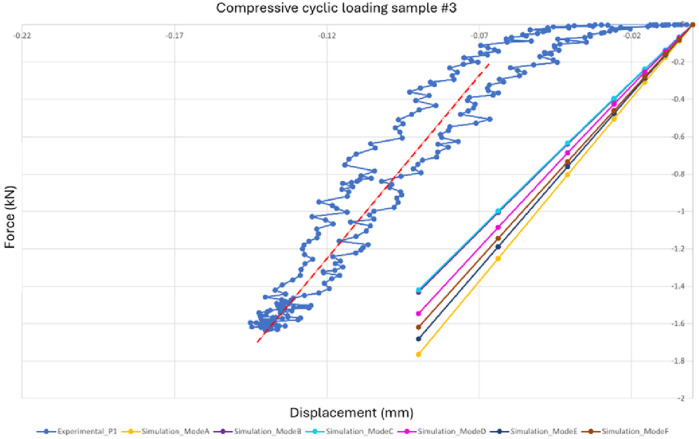
Graph for sample #3 showing the variation in the simulation results with varying boundary conditions compared to the results of Equation 4. The difference between the maximum and minimum slopes by fine-tuning the boundary conditions is as much as 22% (yellow to light blue).

Figure 10 shows how different boundary conditions affect the simulation results in the case of sample #3. The comparison has been made with mode A, which represents the simulation results of the model created using Equation 4 (yellow line). Other boundary conditions that have been considered are mode B (purple line), where there was a top and bottom pad and the contact was defined as general contact, enabling the specification of interactions between multiple or all areas of the model with just one interaction definition (ABAQUS Inc., 2009) mode C (cyan line), where there were still two pads with general contact but specifying that it was a frictionless contact, presuming that the application of ultrasound gel significantly reduces the friction between the pads and the vertebra, and mode D (magenta line), which involved two pads with general contact while implementing a friction coefficient of 0.37 (López-Campos et al., 2018). Mode E (dark blue line) and mode F (brown line) instead just had the top pad and defined a rigid body constraint for the bottom boundary condition. They differ in contact conditions; mode F assumed general contact and frictionless interactions, while mode E also used general contact and a friction coefficient of 0.37.

## 4 Discussion

CT scans are a necessary instrument for the surgeon to understand what is happening under the skin, evaluating the configuration and alignments of bones, for diagnosis and a well informed pre-op planning for surgery as well (Zhu et al., 2023). Having the ability to produce FEA models from scan information in a streamlined way and produce reliable models in an automated surgeon in a dependent but patient-specific mode would be invaluable progress and a prelude to fully IT and AI-driven and robotic surgery practices of the future (Caprara et al., 2021). The present article applied some different methodological process routes to go from scan to biofidelic model on a small number of five vertebrae. The question is if, in such a well-controlled test and scan mode, the modern instruments and processes we have at our disposal can accurately predict the loading behaviour of the samples.

Micro-finite element models are meant to embrace geometry and make it part of the solution, it is after all the scan information that is incorporated into the model (Jones and Wilcox, 2007; Imai, 2015; Franceskides et al., 2020; Garavelli et al., 2025). That is the cornerstone for sample-specific and patient-specific approaches. What is seen here is that there may be reasons why the geometry itself affects the outcomes in ways we cannot yet comprehend. The output showed that even when we tried to create a sample-specific model (as a prelude to a patient-specific model), the relatively behaviour of the modelling process routes we implemented changed and depended on the sample itself. In other words, one would have expected the model output to change, with some models being more accurate than others, but what we saw here is that the rank order of outputs (comparison of model outputs to experiment for the 4 different model process routes) changed along the spine in a gradual way. This is a strong indication that the underlying microgeometry was also involved in the modelling routines.

The present approach had certain limitations, in that it used a small number of samples, a simplified if not simplistic loading configuration, and a simple load measurement parameter outcome (stiffness) to test and compare experiment to model. This was intentional, not a weakness; the aim of this study was to see if in the most rudimentary case a simple potentially operator-free CT-scan-to- FEA model process can be defined. The methodology here was not complicated, in fact very simplified; the FEA models were not finely tuned, in fact, used standard practices and only ironed out the technical issues (Jones and Wilcox, 2007; Imai, 2015; Franceskides et al., 2020; Garavelli et al., 2025) to do with convergences and boundary conditions (which are technical rather than methodological issues). The samples were vertebrae, which were chosen because they combine a good degree of cancellous and cortical bone, can be machined in a chunk-like specimen easy to compress reliably, they have an intricate geometry at the surface and below the surface they also have an internal intricate architecture (to capitalise on the benefits of scan technology) and they are a good representation bone parts of the body that are (1) not easy to access *in-vivo* other than by scanning and (ii) experience asymptomatic fractures indicative of being exactly the bone types one would like to model pre- and post-op. As far as the present ovine vertebrae resemble human ones (which they do in terms of geometry, architecture and basic bone properties) we would expect the present methodology to be applicable to human vertebrae with the same fidelity.

The loading was kept in a simple uniaxial mode because more complex regimens for this small number of samples would have introduced more degrees of freedom and obscured the main scope of the study, which was the different modelling processes and what they offer. Bones are composite structures with viscoelastic properties (Gottesman and Hashin, 1980) and this resulted in a noticeable loop in the force/displacement data; however, the FEA models offer a pseudo-static test prediction, and so the stabilised stiffness in the linear region was used for comparison to model output. As already pointed out, the matching of modelling strategies to a sample changed with each sample and as we went down the spine from L3 to L7. As seen in Figure 1, all samples have relatively similar dimensions, shape, and sizes, excluding any kind of difference due to the minute characteristics of the sample. So, in fact, this offered five variations of a geometrical object design without profound changes in the very geometry of the object. This is a strong point because it allowed for variance at the architecture level without overwhelming changes in the geometry of the object studied.

In the literature, there are several different conversion equations that help convert densities, which can be apparent or ash, to Young’s modulus. The most used and referenced one is the Carter and Hayes (1977) paper, which was one of the earliest and most referenced relationships for predicting Young’s modulus from bone density. An essential consideration when selecting the appropriate equation is the location of the bone and the specific requirements, taking into account whether the bone is subjected solely to compressive force or is also experiencing tensile or torsional forces (Cyganik et al., 2014). For example, the Carter and Hayes (1977) equation, although validated, is based on the behaviours of bones from different pooled sites. This limits its accuracy for precise predictions in specific cases, because the behaviour of the cortical bone changes greatly from that of the cancellous bone. Cyganik et al. (2014) conducted a comparative analysis of five distinct equations, including the Carter and Hayes (1977) equation, focusing on cubes of the femoral heads. They discovered that the equation formulated by Ciarelli et al. (2000), which similarly used cubes from the proximal femora, demonstrated superior precision over the Carter and Hayes (1977) equation. Therefore, the equations formulated by Rho et al. (1995) were selected as they are specifically designed for lumbar vertebrae subjected to compressive loads, assuming that since the vertebrae in this research study are from the same location and with the same loading pattern, they would yield more precise results. Overall, we found that use of modelling processes that are based on Equations 3, 5 (Rho et al., 1995) as given in the literature were more successful than other routes. There may be other more successful attempts in the future, but the purpose of the present efforts was not to solve the entire problem but to contribute towards this lively debate of microfinite element modelling and patient-specific solutions. These are here to stay, but they have limited success, as the present study shows.

A further advantage (other than *in-vivo* scanning) that the m-FEA approaches can handle is that they can capitalise on standing CT scans, an emerging imaging modality that allows the assessment of anatomical structures and pathophysiological conditions in a weight-bearing position. Magnetic resonance imaging (MRI) is another type of medical scan that is routinely used in clinical settings to create detailed images of the inside of the body. Although valuable, especially for its soft tissue contrast, it is not routinely used for orthopaedic cases due to its costs and speed compared to CT or X-ray images, which is why magnetic resonance images are not typically used to construct bone models. Metallic implants also create large magnetic susceptibility differences that cause signal loss and geometric distortion, often rendering the region around a prosthesis unreadable (Germann et al., 2021; Koff et al., 2017). The approach in this research provides insight into how gravity and posture affect various body systems, which is not possible with traditional supine CT scans. For example, Jinzaki et al. (2020) discovered that the volume and shape of the vena cava changed, or the position of the pelvic floor in the body was different if the scanned subject was standing or supine, making CT scans in an upright position key for evaluating and planning surgeries for subjects who have conditions affected by this. Yamada et al. (2020) made a similar discovery but with volumes of the lung and lobes, which are significantly greater in standing position. Generally, standing CT scans take less time than standard CT scans (Jinzaki et al., 2020) and also have a lower radiation dose (Debousset et al., 2005), without compromising image quality, safety and comfort for the patient. The only factor to consider is that some standing CT scanners have support bars that can change the distribution of body weight and reduce it by up to 20% (Shansuddin et al., 2013), which may affect the precision of the weight-bearing evaluation. All of these studies were performed with several different standing CT scanners that were not used clinically.

Some known clinical examples highlight the potential use of the knowledge acquired here for situations where loading and unloading configurations are equally challenging for modelling (i.e., standing CT scans of feet) and we have experience in our labs with at least one particular pathophysiological condition: flatfoot (pes planovalgus), which is encountered at an early age (John et al., 2023; Vergara-Amador et al., 2012; Graham, 2016). Flatfoot does not cause significant musculoskeletal problems, remains asymptomatic in 25% of cases, but causes discomfort and gait and pain problems in later adulthood (Mosca, 2010; Harris and Beath, 1948; Bouchard and Mosca, 2014). Flatfoot can be flexible (FFF) or rigid (RFF), the latter of which is pathological with skeletal and soft tissue problems that require appropriate treatment (Bouchard and Mosca, 2014; Carr et al., 2016; Evans and Rome, 2011). Surgery is a viable option as a means to achieve restoration of normal or near-normal functions of the foot and ankle (Kumar and Sonanis, 2017). Reconstructive surgery involves osteotomies and soft tissue reconstruction to recreate the arch of the foot (Mosca, 2010). However, this is highly subjective and requires the surgeon’s judgement as to how much modelling is appropriate (Mosca, 2014). Complications, such as over and under correction, nerve injury (Tennant et al., 2014), and calcaneocuboid subluxation, are some of the outcomes with complications arising in up to 17.3% of cases (Kumar and Sonanis, 2017). Computer-aided surgical design, such as the use of patient-specific m-FEA, may be a valuable tool to help understand how changing the magnitude and direction of forces alters the distribution of stresses and strains through the bones once the structure of the foot changes due to surgical intervention (Wang et al., 2016). In this instant, it also requires *in-vivo* scans pre- and post-op and in loaded and unloaded bone configuration. Clinical *in-vivo* and standing CT scanners such as the model we used here (HiRise, Curvebeam) are a suitable instrument for this condition, allowing potentially for a more accurate 3D model reconstruction of the foot in this condition.

## 5 Conclusion

Finite element models from living anatomical structures to produce patient-specific models offer improved diagnosis, precision pre-op planning for surgeries, and reliable biofidelic stress loading analysis. In the present study, sheep vertebrae were used to create biofidelic phantoms for scanning by using one of the latest technology high-resolution (300 micron) clinical standing scanners (HiRise, Curvebeam). The fit between the model and the experimental results was best for two equations from the literature given by Rho et al. (1995), while others were less reliable. The selection of the most suitable and universally applicable material conversion equation is significant because it can streamline the route to produce scanner to computer patient-specific models and make this widely available and ultimately more easily obtainable immediately post-scan. The results we obtained showed that, depending on the sample, certain model creation processes were more effective than others. This reinforces the notion that the selection of a material conversion equation should account for the specific bone site from which it was derived, as this can significantly influence the results. Since Rho et al. (1995) defined several equations in their study from different bone sites, we used their suggested relationship for our implementation. The present study could help towards certain clinical conditions where *in-vivo* scanning is required both pre- and post-op and where loading the bones during scanning may also be a prerequisite for a successful modelling and outcome (i.e., flatfoot reconstructive surgeries). In general, our efforts can help validate m-FEA models and their implementation in clinical settings, potentially leading to improved treatment results and a decrease in complications.

## Data Availability

The raw data supporting the conclusions of this article will be made available through the Cranfield University CORD data repository and preservation system at https://cranfield.figshare.com or through one of the authors s.junaid@aston.ac.uk, without undue reservation.
