# Supplementary figures and images for: Producing micro-finite element models from real-time clinical CT scanners: calibration, validation and material mapping strategies
Source: Front Bioeng Biotechnol. 2025 Dec 17;13:1670428. doi: 10.3389/fbioe.2025.1670428 (PMC12754003; doi:10.3389/fbioe.2025.1670428)

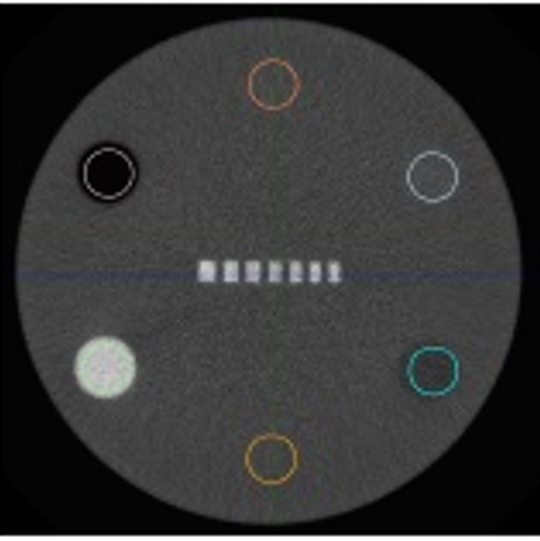

Supplement: Supplementary file 1 [file Image3.jpeg]

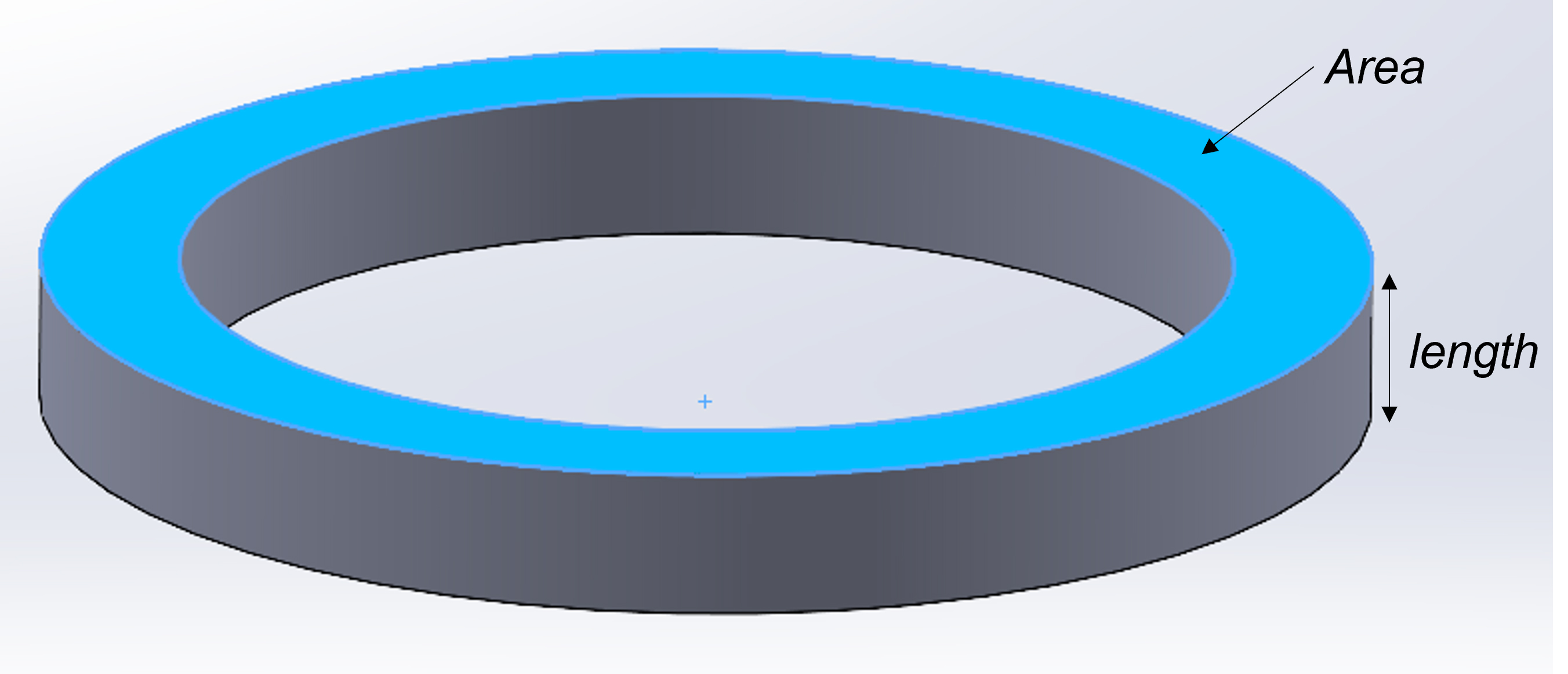

Supplement: Supplementary file 2 [file Image1.jpeg]

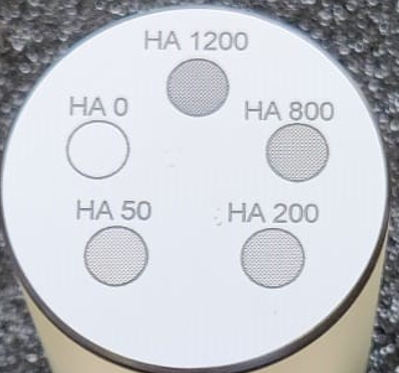

Supplement: Supplementary file 3 [file Image4.jpeg]

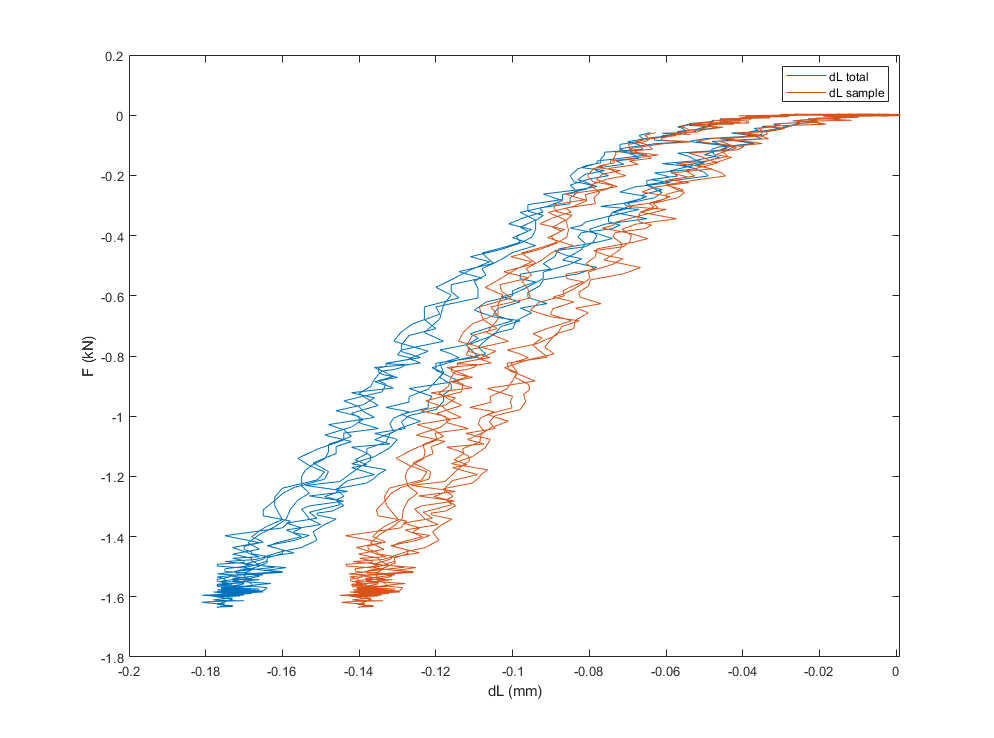

Supplement: Supplementary file 4 [file DataSheet1.zip › Figures/Figure 12.jpg]

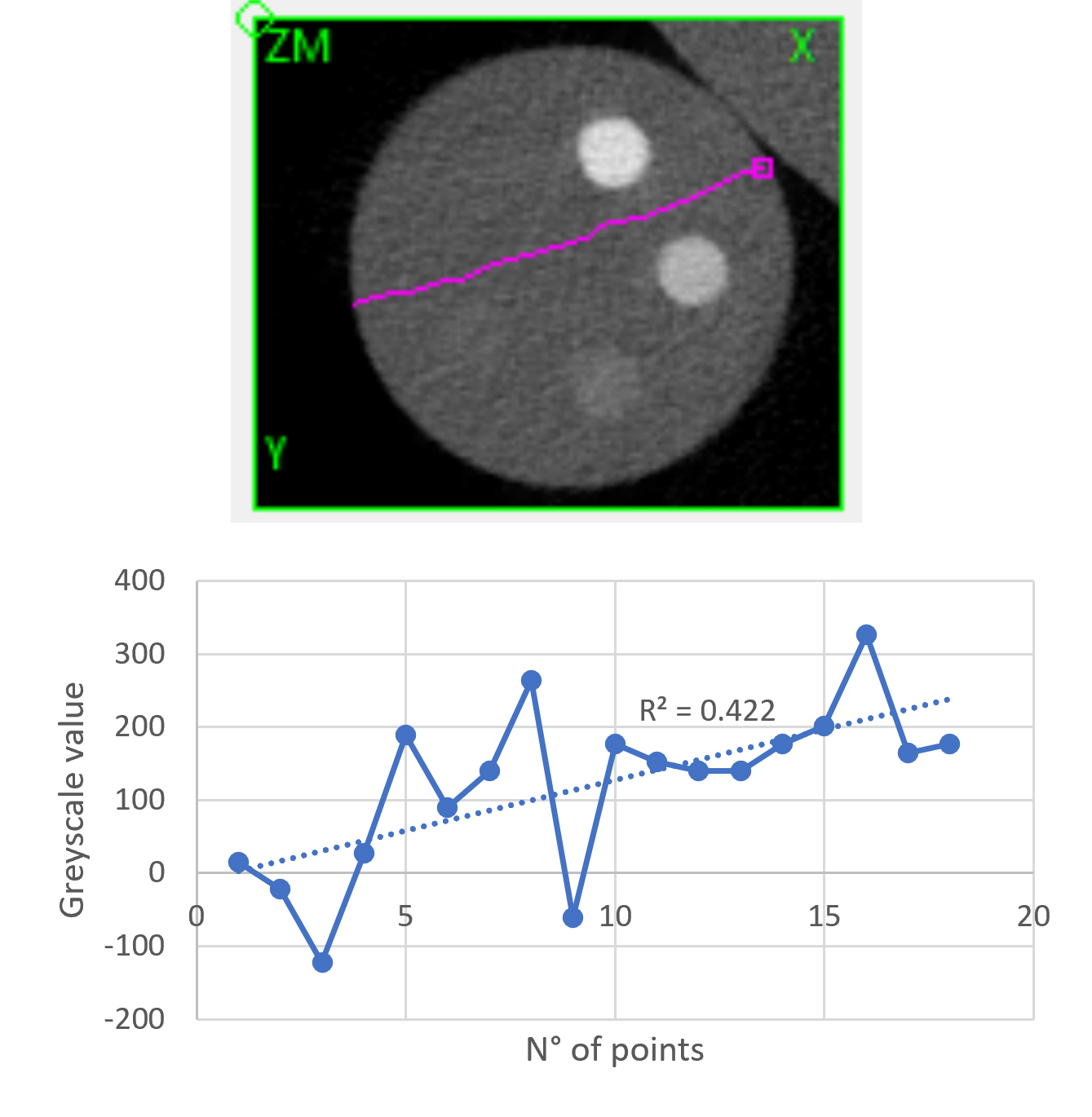

Supplement: Supplementary file 4 [file DataSheet1.zip › Figures/Figure 14(A).jpg]

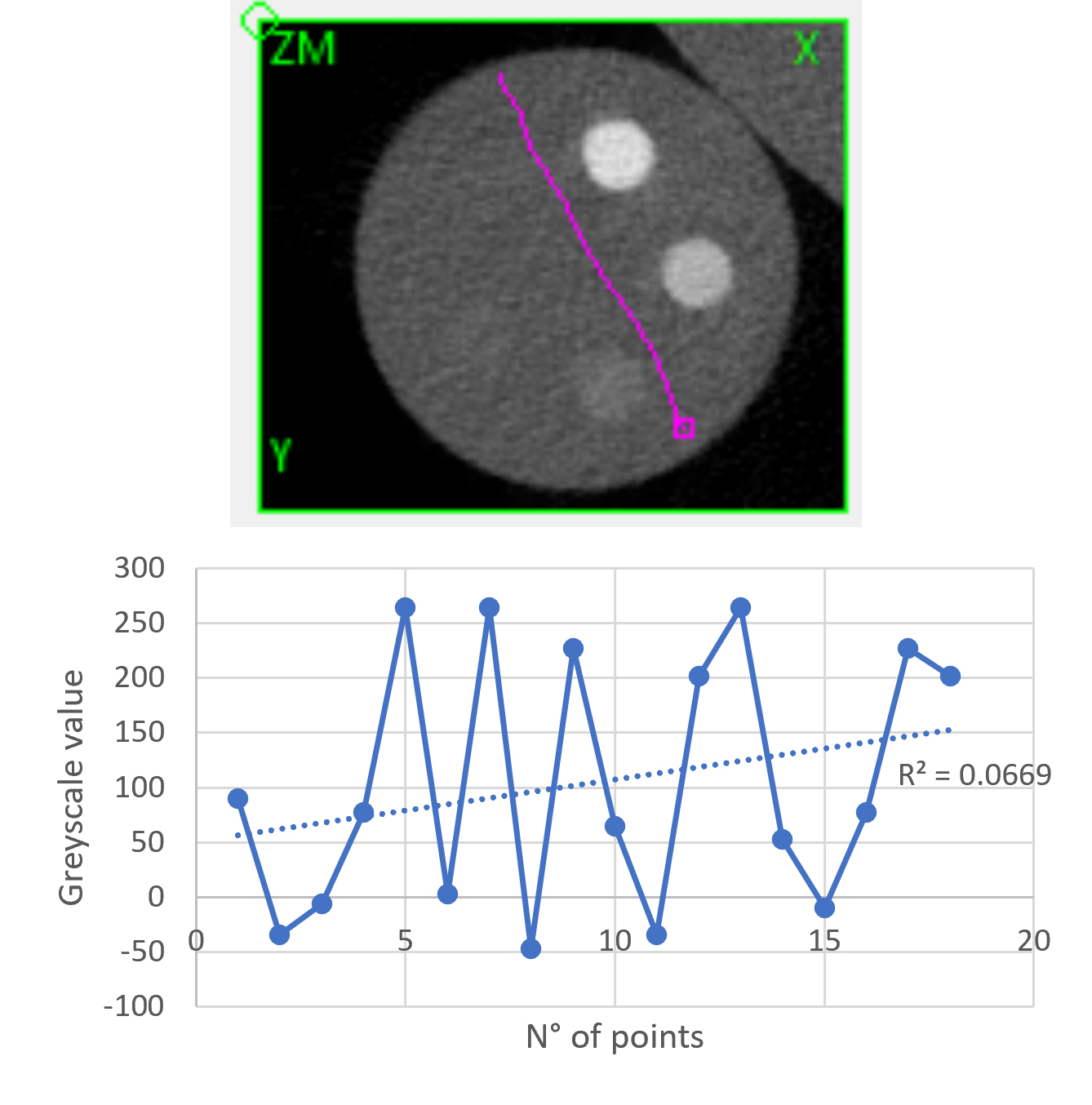

Supplement: Supplementary file 4 [file DataSheet1.zip › Figures/Figure 14(B).jpg]
